# Novel *NPRL3* variant associated with sleep-related hypermotor epilepsy: a case report and educational review

**DOI:** 10.3389/fnins.2025.1669847

**Published:** 2026-01-19

**Authors:** Serena Broggi, Kai-Nicolas Poppert, Matthias Mauritz, Gudrun Kalss, Markus Leitinger, Angela Abicht, Eugen Trinka, Fabio Rossini

**Affiliations:** 1 S.C. Neurology and Stroke Unit, ASST Sette Laghi, Varese, Italy; 2 Department of Neurology, Neurocritical Care, and Neurorehabilitation, Center for Cognitive Neuroscience, Member of European Reference Network EpiCARE, Christian Doppler University Hospital, Paracelsus Medical University, Salzburg, Austria; 3 Neuroscience Institute, Center for Cognitive Neuroscience, Christian Doppler University Hospital, Paracelsus Medical University, Salzburg, Austria; 4 Medical Genetics Center (MGZ), Munich, Germany; 5 Department of Neurology, Friedrich-Baur-Institute, Ludwig-Maximilians-Universität, Munich, Germany; 6 Karl Landsteiner Institute for Clinical Neurosciences, Salzburg, Austria

**Keywords:** paroxysmal nocturnal dystonia, nocturnal seizures, nocturnal frontal lobe epilepsy, personalized medicine, mTOR, hyperkinetic seizure

## Abstract

**Introduction:**

Sleep-related hypermotor epilepsy (SHE) is a rare epileptic syndrome characterized by nocturnal seizures that predominantly arise during sleep, featuring complex motor behaviors. Pathogenic variants in the nitrogen permease regulator-like 3 (*NPRL3*) gene and other regulators of the mTOR pathway have been linked to diverse epilepsy phenotypes, including SHE. SHE is challenging to diagnose due to its diverse presentations, overlap with non-epileptic sleep disorders, and semiological similarities to functional/dissociative seizures (FDS).

**Case report:**

We present the case of a 61-year-old woman with a lifelong history of nocturnal paroxysmal events and focal epilepsy. She experienced stereotyped nocturnal episodes of focal motor seizures with retained consciousness, characterized by hyperkinetic activity and asymmetric posturing. Despite multiple antiseizure medications (ASMs), only carbamazepine (CBZ) provided long-term seizure freedom. Genetic testing revealed a novel heterozygous mutation in the *NPRL3* gene.

**Discussion:**

This case highlights the diagnostic challenges of SHE and the importance of genetic testing in drug-resistant epilepsy. The identified *NPRL3* mutation shows the genetic complexity of SHE and its implications for treatment.

## Introduction

Sleep-related hypermotor epilepsy (SHE) is a rare epileptic syndrome characterized by seizures that occur predominantly during sleep and feature complex, often bizarre, motor behaviors.

Pathogenic variants in the nitrogen permease regulator-like 3 (*NPRL3*) gene, as well as other regulators of the mTOR pathway, have been linked to various epilepsy phenotypes (Ricos et al., 2016), including SHE and familial focal epilepsy with variable foci (FFEVF) (Yin et al., 2023; Yue et al., 2023; Yang D. et al., 2024; Dainelli et al., 2023; Zhang et al., 2023). The NPRL3 gene encodes a protein that is part of the GAP activity toward Rags 1 (GATOR1) complex. The role of the GATOR1 complex is to inhibit the mTOR pathway under unfavorable metabolic conditions. Some variants in the *NPRL3* gene lead to augmented mTOR activation via loss of mTOR inhibition. As a result, these mutations can disrupt normal neuronal activity (including circuitry and excitability), morphology, and architecture, leading to the heterogeneous phenotypic features of these diseases and the variable penetrance observed in affected individuals (Iffland et al., 2022). Historically, SHE has been challenging to diagnose due to its diverse presentations and overlapping semiology, especially with non-epileptic sleep disorders and functional/dissociative seizures (FDS).

We present the case of a 61-year-old woman with a 57-year history of nocturnal paroxysmal events and focal epilepsy, who was found to carry a novel *NPRL3* mutation.

## Case report

A 61-year-old Caucasian woman was admitted to our Video-EEG Monitoring Unit to reevaluate nocturnal paroxysmal events, which had reappeared after a medication switch from carbamazepine (CBZ) to levetiracetam (LEV) after a 26-year seizure-free period. According to the patient and her family, seizure onset occurred before the first year of life. The patient had always experienced highly stereotyped and frequent nocturnal attacks. An unspecified form of focal epilepsy was diagnosed at the age of 4. The history of the patient was unremarkable regarding perinatal complications, early development issues, and acquired brain disorders. There was no family history of epilepsy. The patient was right-handed and had a normal neurological examination. She had to leave school at age 15 due to daytime sleepiness directly linked to the nocturnal attacks but later became a nurse after achieving seizure freedom with CBZ at age 36. Apart from epilepsy, the patient had hypertension, a cleft lip (which had been surgically corrected during childhood), hypothyroidism, and hypercholesterolemia; moreover, the patient experienced a burnout episode approximately 7 years before admission. During a paroxysmal event, the patient described experiencing a tingling sensation in her left foot upon waking from sleep, which then spread throughout her body. This was often accompanied by shortness of breath and a feeling of anxiety. Subsequently, the patient felt her body shake and move uncontrollably. Consciousness was never impaired, and the patient reported always being able to recall the attacks. Sometimes, the tingling was limited to the left foot without further propagation. The duration of the episodes was always described as very brief, lasting approximately 40 s. The patient reported that the attacks initially occurred almost every day for years, with up to 20 seizures per night, without a clear pattern of distribution. There was no history of any other seizure type or status epilepticus. In addition, the patient reported multiple non-seizure-related awakenings from sleep.

The patient had been treated with multiple antiseizure medications (ASMs) since childhood, without significant improvement in seizure frequency. The ASMs used included clonazepam (CLZ), lamotrigine (LTG), nitrazepam, primidone (PRM), valproic acid (VPA), vigabatrin (VGB), phenobarbital (PB), and acetazolamide (ACZ). Seizures ceased only after the initiation of CBZ (reaching a maximum dosage of 1,500 mg/day) at age 36, with the patient remaining seizure-free for the subsequent 26 years. Seizures recurred only when CBZ was switched to LEV due to symptomatic hyponatremia shortly before the current presentation (1,250 mg/day twice a day).

The patient was referred to the Video-EEG Monitoring Unit for evaluation of the reported attacks, with the specific aim of distinguishing epileptic from functional/dissociative seizures. During 5 days of video-EEG monitoring, six stereotyped episodes were recorded. All episodes occurred nocturnally, arising during sleep, specifically immediately following arousals from stage 2 (N2) or stage 3 (N3) of non-rapid eye movement (NREM) sleep.

**Figure 1:**
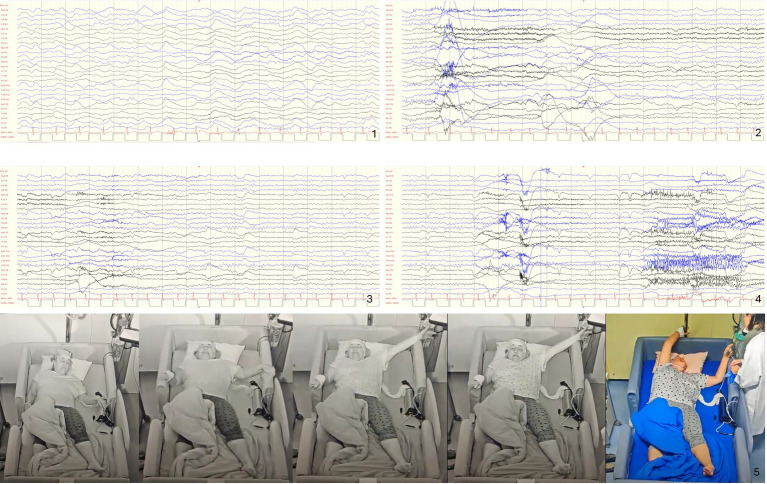
Ictal semiology and EEG: in the example shown, the ictal onset occurs during N3 sleep phase, with 10–12 Hz rhythmic alpha maximal frontally superimposed to the high amplitude delta slow waves (1), from where the patient arouses (2), however before and during (3 and 4) the clinical seizure no apparent ictal pattern is recognizable. Consecutive screenshots with 0.53 Hz high pass and 70 Hz low pass filters, 70 μV/div, 15 s per epoch. (5) The semiology of this typical seizure can be interpreted as a focal preserved consciousness (FPC) seizure, with observable motor manifestations in the form of hyperkinetic behavior and an asymmetric posture, possibly due to propagation from S1 to the symptomatogenic zone in the supplementary sensorimotor area (SSMA) of the frontal lobe (likely lateralized to the right in this case).

Objectively, the episodes were characterized by a dystonic posture of the left leg and foot (tonic extension of the leg and inversion of the foot), sensory symptoms in the form of a pain sensation (possibly linked to the forced and involuntary dystonic posture), abduction and elevation of the left hand with choreoathetoid movements, and intentional “grasping” of nearby objects (e.g., clinging to the bed sides), followed by bilateral, non-integrated unsynchronized hyperkinetic movements of the arms and legs (Figure 1). Subjectively, the patient reported the usual tingling sensation in her left foot upon waking from sleep. The episodes lasted between 3 and 48 s. Ictal EEG showed that the episodes arose shortly after sudden awakening from N2 or N3 sleep stages, accompanied by an arousal and, sometimes, a subsequent transitory bifrontal slowing in the delta range with superimposed alpha activity—possibly representing the scalp signature of an ictal focus too deep to register its faster discharges. However, in any case, a clear ictal correlate on EEG could not be recognized (Figure 1). Ictal ECG consistently showed an increase in heart rate ranging from 20 to 100%. Interictal EEG did not show any epileptiform discharges. Brain MRI [3T, Harness-Protocol (Bernasconi et al., 2019), including post-processing with voxel-based morphometry analysis] was unremarkable. The video-EEG recordings were reviewed in the presence of the patient and her sister, both of whom confirmed their marked similarity to the childhood episodes. Given the strong clinical suspicion for SHE and to define the underlying etiology—despite normal interictal and ictal EEG and non-lesional high-resolution epilepsy protocol MRI—a gene panel targeting genes associated with frontal lobe epilepsies (including *CHRNA2, CHRNA4, CHRNB2, DEPDC5, KCNT1, NPRL3, NPRL2, PRIMA1*, and *PRRT2*) was performed. The analysis identified a novel heterozygous variant in the *NPRL3* gene (NM_001077350.3): c.1283del p.(Val428Alafs*29). According to the American College of Medical Genetics and Genomics/Association for Molecular Pathology (ACMG/AMP) guidelines, this variant was classified as likely pathogenic (where PVS1 and PM2_Supporting denote Pathogenic Very Strong 1 and Pathogenic Moderate 2 Downgraded to Supporting, respectively). The patient had a negative family history. Unfortunately, trio analysis could not be performed due to the unavailability of parental samples. Although a *de novo* origin is suspected, this cannot be definitively confirmed in the absence of parental testing. However, a diagnosis of SHE was established. An initial attempt to slowly switch to lacosamide (LCM) was not tolerated. A switch attempt to eslicarbazepine (ESL) was made. ESL was better tolerated, but the patient failed to achieve seizure freedom. Therefore, the patient is once again receiving treatment with CBZ and remains seizure-free. Modification of the antihypertensive therapy and oral sodium chloride supplementation were necessary to decrease the risk of concurrent hyponatraemia.

## Case discussion

We describe a case of sleep-related hypermotor epilepsy (SHE) in which a definitive diagnosis was only reached after 57 years, following a thorough epilepsy syndrome re-evaluation and modern genetic testing. Since the patient’s history was notable for recent psychiatric disturbances, the seizure recurrence after a long period of seizure freedom, despite being on an appropriate dose of a different ASM, led initially to a suspicion of FDS. However, it is important to point out the red flags that could prompt reconsideration of this interpretation. Stereotyped, brief, and multiple paroxysmal events do not typically occur in the context of FDS, although their presence does not completely rule it out. Similarly, occurrence exclusively during sleep suggests an epileptic seizure rather than FDS, although exceptions have been reported (Higgins and Koutroumanidis, 2022). Semiologically, seizures arising during sleep, with retained consciousness and foot tingling rapidly followed by asymmetric posturing and both integrated and non-integrated hyperkinetic motor manifestations, suggest a symptomatic zone originating in the primary somatosensory cortex (S1) or posterior insula, rapidly propagating to the supplementary motor cortex of the frontal lobe (McGonigal, 2022). This finding is in line with the absence of a clearly epileptiform EEG in our patient and is compatible with the diagnostic criteria for SHE (Riney et al., 2022; Tinuper et al., 2016; Tinuper and Bisulli, 2017). In addition, seizures in the context of SHE are often drug-resistant, but they may respond well to sodium channel blockers, especially CBZ. The reappearance of seizures in this patient after CBZ withdrawal further supports this interpretation. Paroxysmal arousals are also a feature of SHE (Bisulli et al., 2019).

This case emphasizes the importance of genetic clarification in the diagnostic workup of drug-resistant epilepsies, providing a rational basis for a personalized approach to these conditions. The previously unknown variant identified in this patient in the *NPRL3* gene c.1283del p.(Val428Alafs*29) causes a deletion of a nucleotide in the coding region of the gene. This deletion results in a frameshift that alters the protein sequence from amino acid 428, causing the formation of a premature stop codon at position 29, which leads to a truncated, non-functional protein. Given the known role of *NPRL3* in the GAP activity toward Rags 1 (GATOR1) complex and its involvement in mTOR pathway regulation, haploinsufficiency of *NPRL3* represents a biologically plausible pathogenic mechanism. This functional consequence is the reason why the variant meets the PSV1 classification according to the ACMG/AMP guidelines. The likely pathogenic role of this variant is further supported by the PM2_Supporting criterion, which confirms its extreme rarity in the healthy population.

In addition, other truncating NPRL3 variants have been reported in the literature to further support its pathogenicity, including the well-studied founder mutation c.349delG (Iffland et al., 2022), c.1352-4delACAGinsTGACCCATCC (Sim et al., 2016), c.1070delC (Weckhuysen et al., 2016), and c.1522delG (Korenke et al., 2016). The clinical presentation in this patient, consistent with SHE, further supports the relevance of this variant.

For this reason, we decided to reintroduce the sodium channel blocker. Despite the high dosage and the need for sodium supplementation, the patient has good seizure control with monotherapy and reports an improvement in her quality of life.

## Educational review

### General aspects and diagnosis

SHE is a rare focal epileptic syndrome typically manifesting within the first two decades of life, characterized by a stereotyped presentation. Clinical manifestations in SHE predominantly follow arousals from non-rapid eye movement (NREM) sleep, especially stage 1 (N1) or N2, although onset from the N3 sleep stage, as in our patient, has been reported. Seizures tend to frequently cluster nocturnally, which was also a characteristic pattern presented in our case. In the context of seizures arising during sleep, the precise sequence of events is still a matter of debate. Specifically, it is still unclear whether the arousal triggers the seizure or the seizure itself provokes the arousal (ictal arousal/awakening). On the other hand, paroxysmal arousals can be interpreted as hypnopompic seizures, as described by Awad and Lüders (2010), where the arousal itself is the main clinical manifestation of the seizure.

The diagnosis of SHE relies solely on clinical criteria, emphasizing the importance of recognizing its core features: onset during sleep, stereotypical semiology, and abrupt transitions. Detailed diagnostic criteria are provided in Table 1 (Riney et al., 2022; Tinuper et al., 2016; Tinuper and Bisulli, 2017). Individuals with SHE may experience a high seizure frequency, leading to sleep fragmentation and daytime consequences such as excessive sleepiness and fatigue, as well as cognitive deficits and psychiatric comorbidities. Seizures exhibit an abrupt onset and offset, are of short duration, and often entail preserved consciousness. Seizure semiology is typically hyperkinetic, which includes asymmetric dystonic or tonic postures or violent, often bizarre, asynchronous movements of the limbs and trunk. Seizures with paroxysmal arousals (SPAs), characterized by paroxysmal arousals without more apparent motor clinical features, are also a typical feature of the syndrome (Loddo et al., 2020). Our patient’s presentation is a textbook illustration of these core features.

**Table 1:** Diagnostic criteria of SHE, adapted from Riney et al. (2022), Tinuper et al. (2016), and Tinuper and Bisulli (2017).

Clinical features	SHE is characterized by the occurrence of brief (<2 min) seizures with stereotyped motor patterns within individuals and abrupt onset and offset.
	The most common clinical expression consists of “hypermotor” events.
	Seizures of SHE occur predominantly during sleep; however, seizures during wakefulness may also occur.
Electroclinical features	Interictal and ictal scalp EEG features may be uninformative.
	Prolonged video-EEG recording is the best available diagnostic test to assess the occurrence of seizures but, if negative, does not rule out the diagnosis.
	Sleep-related hypermotor seizures may arise from various frontal as well as extrafrontal areas.
Diagnostic certainty	Diagnosis of SHE is primarily based on clinical history. The absence of clear interictal and ictal EEG correlates, both during wakefulness and sleep, does not exclude the diagnosis.
	Certainty of diagnosis can be categorized into 3 levels: witnessed (possible) SHE, video documented (clinical) SHE, and video-EEG documented (confirmed) SHE.
Etiology	In a majority of patients, the etiology is unknown.
	Identified etiologies are heterogeneous and include structural anomalies such as focal cortical dysplasia, acquired injuries, and genetic causes.
	No specific clinical features distinguish etiologies.

Video-EEG is the gold standard for the diagnosis of SHE. However, the absence of an ictal EEG pattern during video monitoring does not exclude the diagnosis, as illustrated in our case. Differential diagnoses include NREM parasomnias (Moro et al., 2023; Proserpio et al., 2019; Loddo et al., 2020) and functional/dissociative seizures (FDS) (Wan et al., 2021). However, unlike patients with SHE, patients with NREM parasomnias have no memory of the incident. Furthermore, NREM parasomnias often resolve with age (Tinuper and Bisulli, 2017).

The seizure onset zone is often, but not exclusively, frontal; extrafrontal onsets, such as in our patient, have been documented using imaging, including ictal source imaging, and stereo-EEG in pre-surgical and surgical cohorts. These include the temporal, parietal, insular, and occipital cortices (Tinuper and Bisulli, 2017; Gibbs et al., 2015; Gibbs et al., 2018; Nobili et al., 2004; Wadi et al., 2024; Proserpio et al., 2011; Gibbs et al., 2019; Arbune et al., 2020; Montavont et al., 2013; Dobesberger et al., 2008).

### Etiology and physiopathology

The etiology of SHE is primarily genetic, including genetically determined structural abnormalities, such as focal cortical dysplasias (FCDs) (Tassi et al., 2012; Iffland et al., 2022). Over the years, pathogenic variants in different genes have been associated with the condition (Licchetta et al., 2020; Bisulli et al., 2019; Arenas-Cabrera et al., 2022; Wan et al., 2021; Yang Y. et al., 2024).

These include genes encoding neurotransmitter receptors and regulators, such as *CHRNA4* and *CHRNB2, CHRNA2* (encoding nAChR subunits), *PRIMA1* (encoding a protein that anchors acetylcholinesterase, which is crucial for terminating Ach-mediated signal at the synapse), and *GABRG2* (encoding the gamma-2 subunit of the GABA-A receptor). Other implicated genes include those encoding ion channels, such as *KCNT1* (encoding a sodium-activated potassium channel subunit), as well as genes encoding components of the mTOR cascade pathway, such as *DEPDC5*, *NPRL2, NPRL3*, *TSC1*, and *PAK3* (Gambardella et al., 2025). In addition, SHE has been reported in patients with mucopolysaccharidoses (MPS) (Bonanni et al., 2014; Abramova et al., 2021) and aspartylglucosaminuria (Ambrosetto and Santucci, 2009). This heterogeneity underscores the genetic complexity of the disorder.

Family history is present in approximately 25% of cases, with rare instances of autosomal dominant SHE (ADSHE) (Kurahashi and Hirose, 2002), previously known as autosomal dominant nocturnal frontal lobe epilepsy (ADNFLE) (Scheffer et al., 1995; Villa et al., 2022). ADNFLE was the first ever described genetic cause of an epileptic syndrome and has been linked to pathogenic variants in the *CHRNA4* gene (Cholinergic Receptor Nicotinic Alpha 4 Subunit MIM *118504), exhibiting an autosomal dominant pattern of transmission.

### A focus on mTORopathies and NPRL3

The mTOR signaling pathway encompasses the mTOR cascade, regulating cellular growth, proliferation, and migration in response to the presence of diverse substrates, including amino acids, insulin, growth factors, and oxygen. Disorders resulting from mutations in genes encoding components of the mTOR cascade are termed “mTORopathies.” These mutations commonly result in aberrant activation of the mTOR signaling pathway, which, in turn, causes irregular neuronal morphology, cortical laminar structure alterations, and increased neuronal excitability (Baldassari et al., 2016). *NPRL3* is a highly conserved gene located on chromosome 6p13.3 and encodes nitrogen permease regulator-like 3 (*NPRL3*), a protein containing 569 amino acids that is a component of the GAP activity toward Rags 1 (GATOR1) complex, an inhibitor of the mTOR pathway. Mutations in the *NPRL3* gene have been associated with epilepsy with and without malformations of cortical development (MCD), leading to hyperactivation of the mTOR pathway (Weckhuysen et al., 2016) and decoupling mTOR activation from the metabolic state. As a result, epilepsy caused by GATOR1 complex mutations typically exhibits a focal pattern and may be linked to MCD (displaying diverse phenotypes) or a lowered seizure threshold with non-lesional MRI (Iffland et al., 2019; Yin et al., 2023). The proportion of individuals with MCD among those carrying *NPRL3* mutations varies across studies. Research on a founder *NPRL3* pedigree among Old Order Mennonites, dating back to 1727, reported heterogeneous clinical phenotypes, including brain imaging abnormalities such as polymicrogyria and FCD, with incomplete epilepsy penetrance of 28% (Iffland et al., 2022). Among patients with MCD, approximately 62.5% of those with *NPRL3*-related epilepsy exhibited FCD. In the same study, non-lesional MRI was reported in approximately 70% of patients with *NPRL3*-related epilepsy. Interestingly, there was no association between MRI results and epilepsy severity, drug resistance, prevalence, and outcomes. Another study identified pathogenic *NPRL3* variants in patients with FCD, suggesting *NPRL3* as a causal variant in these cases. In addition, a study on Chinese children with *NPRL3*-related epilepsy found that 62.5% of patients presented with FCD, the most common neuroimaging abnormality (Sim et al., 2016).

In a case series of two pediatric patients with novel *NPRL3* variants, along with a literature review (Yang D. et al., 2024) of 114 cases of *NPRL3*-related epilepsy, the majority of patients carried nonsense, missense, and frameshift variants leading to loss of function. Although a clear genotype–phenotype correlation was not postulated, both the patient data and literature review suggested that drug-resistant epilepsy is more frequent in patients carrying nonsense variants, missense variants, and exonic deletions (Yang Y. et al., 2024). However, only 12 of the 116 patients had SHE, and the data refer to the entire cohort.

### Therapy

Randomized clinical trials (RCTs) assessing pharmacological or non-pharmacological interventions for SHE are currently unavailable. Pharmacological therapy for SHE traditionally involves CBZ as the first-line treatment, with topiramate, lacosamide, and acetazolamide considered as add-on strategies for unsatisfactory responses to monotherapy (Asioli et al., 2020). SHE associated with pathogenic variants in the subunits of n-acetylcholine receptors (nAChRs) has been reported to be responsive to nicotine exposure (Becchetti et al., 2020; Fox et al., 2021; Brodtkorb et al., 2021; Nguyen et al., 2021; Lossius et al., 2020; Willoughby et al., 2003). However, despite active pharmacological interventions, achieving satisfactory seizure control remains difficult in patients with SHE, with approximately one-third being drug-resistant (Asioli et al., 2020).

In cases of drug-resistant epilepsy, surgical treatment stands as a significant treatment option, especially when MCD are recognized or suspected through pre-surgical evaluation (Kumar et al., 2018; Menghi et al., 2018; Wan et al., 2021; Zhang et al., 2023).

Lastly, as with other mTORopathies, when surgery is not considered an option, a recent non-controlled study investigated the role of everolimus in treating MRI-negative *NPRL2*- and *NPRL3*-associated epilepsies. Everolimus is an mTOR inhibitor that directly targets the underlying pathway dysfunction responsible for neuronal hyperexcitability and may help control seizures arising from subclinical dysplasias that are not detectable by current MRI technology. The study showed very promising results in terms of seizure reduction and seizure freedom; however, all patients experienced adverse events that led to dose reduction or even drug discontinuation (Carapancea et al., 2025).

## Conclusion

This case underscores the genetic complexity of SHE and highlights the necessity and value of prolonged video-EEG monitoring and thorough genetic testing in patients with atypical or drug-resistant epilepsy. Understanding the heterogeneous genetic basis of SHE not only aids in identifying affected individuals and differentiating individuals with epilepsy from those with non-epileptic spells but also plays a crucial role in genetic counseling, guiding appropriate treatment selection, and advancing knowledge of the disorder’s pathogenesis.

## References

[ref1] Abramova A. A. Attarian H. P. Dolgova S. M. Belyakova-Bodina A. I. Iakovenko E. V. Broutian A. G. (2021). Sleep-related hypermotor epilepsy in a patient with mucopolysaccharidosis type III. Sleep Sci 14, 97–100. doi: 10.5935/1984-0063.20200113, 34917281 PMC8663726

[ref2] Ambrosetto G. Santucci M. (2009). Sleep-related hypermotor seizures in aspartylglucosaminuria: a case report. Epilepsia 50, 1638–1640. doi: 10.1111/j.1528-1167.2008.01991.x, 19175389

[ref3] Arbune A. A. Nikanorova M. Terney D. Beniczky S. (2020). REM-sleep related hypermotor seizures: video documentation and ictal source imaging. Brain Dev. 42, 503–507. doi: 10.1016/j.braindev.2020.04.003, 32340922

[ref4] Arenas-Cabrera C. Baena-Palomino P. Sánchez-García J. Oliver-Romero M. Chocrón-González Y. Caballero-Martínez M. (2022). Sleep-related hypermotor epilepsy with genetic diagnosis: description of a case series in a tertiary referral hospital. J. Central Nervous Syst. Dis. 14. doi: 10.1177/11795735211060114, 35177946 PMC8844731

[ref5] Asioli G. M. Rossi S. Bisulli F. Licchetta L. Tinuper P. Provini F. (2020). Therapy in sleep-related hypermotor epilepsy (SHE). Curr. Treat. Options Neurol. 22, 1–13. doi: 10.1007/S11940-020-0610-1, 31997091

[ref6] Awad A. M. Lüders H. O. (2010). Hypnopompic seizures. Epileptic Disord. 12, 270–274. doi: 10.1684/epd.2010.0336, 21030341

[ref7] Baldassari S. Licchetta L. Tinuper P. Bisulli F. Pippucci T. (2016). GATOR1 complex: the common genetic actor in focal epilepsies. J. Med. Genet. 53, 503–510. doi: 10.1136/jmedgenet-2016-103883, 27208208

[ref8] Becchetti A. Grandi L. C. Colombo G. Meneghini S. Amadeo A. (2020). Nicotinic receptors in sleep-related hypermotor epilepsy: pathophysiology and pharmacology. Brain Sci. 10:907. doi: 10.3390/brainsci10120907, 33255633 PMC7761363

[ref9] Bernasconi A. Cendes F. Theodore W. H. Gill R. S. Koepp M. J. Hogan R. E. . (2019). Recommendations for the use of structural magnetic resonance imaging in the care of patients with epilepsy. Epilepsia 60, 1054–1068. doi: 10.1111/epi.15612 31135062

[ref10] Bisulli F. Licchetta L. Tinuper P. (2019). Sleep-related hypermotor epilepsy (SHE): a unique syndrome with heterogeneous genetic etiologies. Sleep Sci. Pract. 3. doi: 10.1186/S41606-019-0035-5

[ref11] Bonanni P. Volzone A. Randazzo G. Antoniazzi L. Rampazzo A. Scarpa M. . (2014). Nocturnal frontal lobe epilepsy in mucopolysaccharidosis. Brain Dev. 36, 826–829. doi: 10.1016/j.braindev.2013.12.002, 24447995

[ref12] Brodtkorb E. Myren-Svelstad S. Knudsen-Baas K. M. Nakken K. O. Spigset O. (2021). Precision treatment with nicotine in autosomal dominant sleep-related hypermotor epilepsy (ADSHE): An observational study of clinical outcome and serum cotinine levels in 17 patients. Epilepsy Res. 178:106792. doi: 10.1016/j.eplepsyres.2021.106792, 34763266

[ref13] Carapancea E. Eklund E. A. Verhelst H. Cilio M. R. (2025). Everolimus precision therapy in NPRL2- and NPRL3-related epilepsy. Epilepsia 66, e219–e225. doi: 10.1111/epi.18543, 40956028

[ref14] Dainelli A. Iacomino M. Rossato S. Bugin S. Traverso M. Severino M. . (2023). Refining the electroclinical spectrum of NPRL3-related epilepsy: a novel multiplex family and literature review. Epilepsia Open 8, 1314–1330. doi: 10.1002/epi4.12798, 37491868 PMC10690669

[ref15] Dobesberger J. Ortler M. Unterberger I. Walser G. Falkenstetter T. Bodner T. . (2008). Successful surgical treatment of insular epilepsy with nocturnal hypermotor seizures. Epilepsia 49, 159–162. doi: 10.1111/j.1528-1167.2007.01426.x, 18028409

[ref16] Fox J. Thodeson D. M. Dolce A. M. (2021). Nicotine: a targeted therapy for epilepsy due to nAChR gene variants. J. Child Neurol. 36, 371–377. doi: 10.1177/0883073820974851, 33284031

[ref17] Gambardella A. Liu Y.‐. C. Bennett M. F. Green T. E. Damiano J. A. Fortunato F. . (2025). PAK3 pathogenic variant associated with sleep-related hypermotor epilepsy in a family with parental mosaicism. Epilepsia Open 10, 593–601. doi: 10.1002/epi4.13124, 39806575 PMC12014923

[ref18] Gibbs S. A. Figorilli M. Casaceli G. Proserpio P. Nobili L. (2015). Sleep related hypermotor seizures with a right parietal onset. J. Clin. Sleep Med. 11, 953–955. doi: 10.5664/jcsm.4952, 25902821 PMC4513272

[ref19] Gibbs S. A. Proserpio P. Francione S. Mai R. Cardinale F. Sartori I. . (2019). Clinical features of sleep-related hypermotor epilepsy in relation to the seizure-onset zone: a review of 135 surgically treated cases. Epilepsia 60, 707–717. doi: 10.1111/epi.14690, 30866067

[ref20] Gibbs S. A. Proserpio P. Francione S. Mai R. Cossu M. Tassi L. . (2018). Seizure duration and latency of hypermotor manifestations distinguish frontal from extrafrontal onset in sleep-related hypermotor epilepsy. Epilepsia 59, e130–e134. doi: 10.1111/EPI.14517, 30009443

[ref21] Higgins S. Koutroumanidis M. (2022). Psychogenic non-epileptic seizures arising almost exclusively from sleep. Seizure 99, 43–47. doi: 10.1016/j.seizure.2022.05.001 35588647

[ref22] Iffland P. H. 2nd Carson V. Bordey A. Crino P. B. (2019). GATORopathies: the role of amino acid regulatory gene mutations in epilepsy and cortical malformations. Epilepsia 60, 2163–2173. doi: 10.1111/epi.16370, 31625153 PMC7155771

[ref23] Iffland P. H. Everett M. E. Cobb-Pitstick K. M. Bowser L. E. Barnes A. E. Babus J. K. . (2022). NPRL3 loss alters neuronal morphology, mTOR localization, cortical lamination and seizure threshold. Brain 145, 3872–3885. doi: 10.1093/brain/awac044, 35136953 PMC10200289

[ref26] Korenke G. C. Eggert M. Thiele H. Nürnberg P. Sander T. Steinlein O. K. (2016). Nocturnal frontal lobe epilepsy caused by a mutation in the GATOR1 complex gene NPRL3. Epilepsia 57, e60–e63. doi: 10.1111/epi.13307 26786403

[ref27] Kumar J. Solaiman A. Mahakkanukrauh P. Mohamed Rashidi P. M. Das S. (2018). Sleep related epilepsy and pharmacotherapy: an insight. Front. Pharmacol. 9:1088. doi: 10.3389/FPHAR.2018.01088 30319421 PMC6171479

[ref28] Kurahashi H. Hirose S. (2002). “Autosomal dominant sleep-related hypermotor (hyperkinetic) epilepsy” in GeneReviews® (Seattle: University of Washington). 20301348

[ref29] Licchetta L. Pippucci T. Baldassari S. Minardi R. Provini F. Mostacci B. . (2020). Sleep-related hypermotor epilepsy (SHE): contribution of known genes in 103 patients. Seizure 74, 60–64. doi: 10.1016/J.SEIZURE.2019.11.009, 31835056

[ref30] Loddo G. Baldassarri L. Zenesini C. Licchetta L. Bisulli F. Cirignotta F. . (2020). Seizures with paroxysmal arousals in sleep-related hypermotor epilepsy (SHE): dissecting epilepsy from NREM parasomnias. Epilepsia 61, 2194–2202. doi: 10.1111/EPI.16659, 32949468

[ref31] Lossius K. de Saint Martin A. Myren-Svelstad S. Bjørnvold M. Minken G. Seegmuller C. . (2020). Remarkable effect of transdermal nicotine in children with CHRNA4-related autosomal dominant sleep-related hypermotor epilepsy. Epilepsy Behav. 105:106944. doi: 10.1016/j.yebeh.2020.106944, 32097883

[ref32] McGonigal A. (2022). Frontal lobe seizures: overview and update. J. Neurol. 269, 3363–3371. doi: 10.1007/s00415-021-10949-0 35006387

[ref33] Menghi V. Bisulli F. Tinuper P. Nobili L. (2018). Sleep-related hypermotor epilepsy: prevalence, impact and management strategies. Nat. Sci. Sleep 10, 317–326. doi: 10.2147/NSS.S152624, 30349413 PMC6186898

[ref34] Montavont A. Kahane P. Catenoix H. Ostrowsky-Coste K. Isnard J. Guénot M. . (2013). Hypermotor seizures in lateral and mesial parietal epilepsy. Epilepsy Behav. 28, 408–412. doi: 10.1016/j.yebeh.2013.05.030, 23872083

[ref35] Moro M. Pastore V. P. Marchesi G. Proserpio P. Tassi L. Castelnovo A. . (2023). Automatic video analysis and classification of sleep-related hypermotor seizures and disorders of arousal. Epilepsia 64, 1653–1662. doi: 10.1111/epi.17605, 37013671

[ref36] Nguyen S. M. Deering L. Nelson G. T. McDaniel S. S. (2021). Nicotine patch improved autosomal dominant sleep-related hypermotor epilepsy. Pediatr. Neurol. 123, 41–42. doi: 10.1016/j.pediatrneurol.2021.07.006, 34392009

[ref37] Nobili L. Cossu M. Mai R. Tassi L. Cardinale F. Castana L. . (2004). Sleep-related hyperkinetic seizures of temporal lobe origin. Neurology 62, 482–485. doi: 10.1212/01.WNL.0000106945.68292.DC, 14872038

[ref38] Proserpio P. Cossu M. Francione S. Tassi L. Mai R. Didato G. . (2011). Insular-opercular seizures manifesting with sleep-related paroxysmal motor behaviors: a stereo-EEG study. Epilepsia 52, 1781–1791. doi: 10.1111/j.1528-1167.2011.03254.x, 21883183

[ref39] Proserpio P. Loddo G. Zubler F. Ferini-Strambi L. Licchetta L. Bisulli F. . (2019). Polysomnographic features differentiating disorder of arousals from sleep-related hypermotor epilepsy. Sleep 42:zsz166. doi: 10.1093/SLEEP/ZSZ166, 31609388

[ref40] Ricos M. G. Hodgson B. L. Pippucci T. Saidin A. Ong Y. S. Heron S. E. . (2016). Mutations in the mammalian target of rapamycin pathway regulators NPRL2 and NPRL3 cause focal epilepsy. Ann. Neurol. 79, 120–131. doi: 10.1002/ana.24547, 26505888

[ref41] Riney K. Bogacz A. Somerville E. Hirsch E. Nabbout R. Scheffer I. E. . (2022). International league against epilepsy classification and definition of epilepsy syndromes with onset at a variable age. Epilepsia 63, 1443–1474. doi: 10.1111/epi.17240 35503725

[ref42] Scheffer I. E. Bhatia K. P. Lopes-Cendes I. Fish D. R. Marsden C. D. Andermann E. . (1995). Autosomal dominant nocturnal frontal lobe epilepsy. A distinctive clinical disorder. Brain 118, 61–73. doi: 10.1093/brain/118.1.61 7895015

[ref43] Sim J. C. Scerri T. Fanjul-Fernández M. Riseley J. R. Gillies G. Pope K. . (2016). Familial cortical dysplasia caused by mutation in the mammalian target of rapamycin regulator NPRL3. Ann. Neurol. 79, 132–137. doi: 10.1002/ana.24502 26285051

[ref44] Tassi L. Garbelli R. Colombo N. Bramerio M. Russo G. L. Mai R. . (2012). Electroclinical, MRI and surgical outcomes in 100 epileptic patients with type II FCD. Epileptic Disord. 14, 257–266. doi: 10.1684/epd.2012.0525, 22963868

[ref45] Tinuper P. Bisulli F. (2017). From nocturnal frontal lobe epilepsy to sleep-related hypermotor epilepsy: a 35-year diagnostic challenge. Seizure Eur. J. Epilepsy. 44, 87–92. doi: 10.1016/j.seizure.2016.11.023 28027860

[ref46] Tinuper P. Bisulli F. Cross J. H. Hesdorffer D. Kahane P. Nobili L. . (2016). Definition and diagnostic criteria of sleep-related hypermotor epilepsy. Neurology 86, 1834–1842. doi: 10.1212/WNL.0000000000002666, 27164717 PMC4862248

[ref47] Villa C. Arrigoni F. Rivellini E. Lavitrano M. de Gioia L. Ferini-Strambi L. . (2022). Exome sequencing in an ADSHE family: VUS identification and limits. Int. J. Environ. Res. Public Health 19:12548. doi: 10.3390/ijerph191912548, 36231847 PMC9565017

[ref48] Wadi L. Khweileh M. Agashe S. Southwell D. Parikh P. Frauscher B. (2024). Sleep-related hypermotor seizures originating from the occipital lobe. Epileptic Disord. 26, 868–874. doi: 10.1002/epd2.20285, 39305462

[ref49] Wan H. Wang X. Chen Y. Jiang B. Chen Y. Hu W. . (2021). Sleep-related hypermotor epilepsy: etiology, electro-clinical features, and therapeutic strategies. Nat. Sci. Sleep 13, 2065–2084. doi: 10.2147/NSS.S330986, 34803415 PMC8598206

[ref50] Weckhuysen S. Marsan E. Lambrecq V. Marchal C. Morin-Brureau M. An-Gourfinkel I. . (2016). Involvement of GATOR complex genes in familial focal epilepsies and focal cortical dysplasia. Epilepsia 57, 994–1003. doi: 10.1111/epi.13391, 27173016

[ref51] Willoughby J. O. Pope K. J. Eaton V. (2003). Nicotine as an antiepileptic agent in ADNFLE: an N-of-one study. Epilepsia 44, 1238–1240. doi: 10.1046/j.1528-1157.2003.58102.x-i1, 12919397

[ref52] Yang Y. Tuo J. Zhang J. Xu Z. Luo Z. (2024). Pathogenic genes implicated in sleep-related hypermotor epilepsy: a research progress update. Front. Neurol. 15:1416648. doi: 10.3389/fneur.2024.1416648, 38966089 PMC11222571

[ref53] Yang D. Wang J. Qin Z. Feng J. Mao C. Chen Y. . (2024). Phenotypic and genotypic characterization of NPRL3-related epilepsy: two case reports and literature review. Epilepsia Open 9, 33–40. doi: 10.1002/epi4.12856 37902097 PMC10839296

[ref25] Yin K. Lei X. Yan Z. Yang Y. Deng Q. Lu Q. . (2023). Clinical and genetic features of GATOR1 complex-associated epilepsy. Journal of medical genetics. 60, 784–790. doi: 10.1136/jmg-2021-108364 36604176

[ref54] Yue P. Wang P. Yu G. Zhu X. Wu D. Ding Z. . (2023). The clinical features of familial focal epilepsy with variable foci and NPRL3 gene variant. PLoS One. doi: 10.1371/journal.pone.0284924 PMC10132533 37099548

[ref55] Zhang H. Deng J. Wang X. Chen C. Chen S. Dai L. . (2023). Clinical phenotypic and genotypic characterization of NPRL3-related epilepsy. Front. Neurol. 14:1113747. doi: 10.3389/fneur.2023.1113747 36937533 PMC10018541

